# Anti-inflammatory diets for prediabetes remission: a mechanistic and practical roadmap

**DOI:** 10.3389/fimmu.2026.1798089

**Published:** 2026-04-01

**Authors:** Qian Liu, Jiale Zhang, Hongwei Guo, Jianfeng Liu

**Affiliations:** 1 Heilongjiang University of Chinese Medicine, Harbin, China; 2 China Science and Technology Development Center for Chinese Medicine, Beijing, China; 3 Institute for the History of Chinese Medicine and Medical Literature, China Academy of Chinese Medical Sciences, Beijing, China

**Keywords:** anti-inflammatory, anti-inflammatory diets, DIETS, prediabetes, T2DM

## Abstract

Prediabetes affects a significant portion of the global population. Shifting the clinical paradigm from delaying type 2 diabetes (T2D) to actively achieving remission offers a transformative opportunity. This perspective article posits that anti-inflammatory dietary patterns are a cornerstone strategy for prediabetes remission. We explore the mechanistic pathways through which diet-driven inflammation modulates glycemic control and argue for the adoption of the Dietary Inflammatory Index (DII) as a practical, integrative metric to guide dietary choices. By synthesizing evidence from major clinical trials and aligning with the recently released 2025-2030 Dietary Guidelines for Americans, we propose a scalable implementation framework. This article presents our position that remission should be treated as a distinct clinical endpoint, with diet quality, specifically its inflammatory potential, serving as a key modifiable target. We discuss the scientific rationale, propose practical tools for clinical application, and critically acknowledge the limitations of the current evidence base, emphasizing the need for future research to refine and personalize this approach.

## Introduction

Prediabetes, as a critical precursor to type 2 diabetes (T2D) and cardiovascular diseases, impacts over one-third of adults worldwide (1). For decades, clinical management has focused primarily on delaying progression to overt diabetes through modest lifestyle changes or pharmacotherapy aimed at risk factor control. However, recent research confirms that remission—defined as a sustained return to normal glucose regulation (fasting glucose <100 mg/dL, 2-hour glucose post-oral glucose tolerance test <140 mg/dL, and HbA1c <5.7%) without glucose-lowering medications—is an achievable therapeutic goal, offering a transformative opportunity to interrupt the trajectory toward T2D and its long-term complications (2–5). This paradigm shift from risk mitigation to active reversal is grounded in a deeper understanding of prediabetes pathophysiology. At the heart of prediabetes lies chronic low-grade inflammation, a systemic process that drives insulin resistance, impairs β-cell function, and perpetuates metabolic dysfunction (6). Pro-inflammatory diets—typically high in refined carbohydrates, processed meats, and saturated fats—exacerbate this inflammatory milieu by elevating circulating cytokines and promoting oxidative stress. In contrast, anti-inflammatory dietary patterns, characterized by abundant fiber, polyphenols, and omega-3 fatty acids from whole plant foods, counteract these processes through multiple pathways, including gut microbiota modulation, reduced visceral adipose inflammation, and enhanced insulin signaling. Therefore, establishing anti-inflammatory dietary patterns as the cornerstone strategy for prediabetes remission holds strong scientific merit. In this article, we propose that the Dietary Inflammatory Index (DII)—a validated tool that scores the inflammatory potential of a diet—serves as a useful integrative marker for capturing this diet-inflammation relationship. We further suggest that the recently released 2025–2030 Dietary Guidelines for Americans (DGA) provide a practical, though not specific, framework for implementing low-DII dietary patterns at scale (7, 8). By integrating mechanistic insights with practical, evidence-based guidance, this approach shifts prediabetes care from mere delay to proactive reversal, providing a scalable framework for both clinical practice and public health.

## Our perspective: inflammation-targeted remission as a clinical endpoint

As an opinion piece, this article aims to clearly articulate a distinct position on prediabetes management. This article argues for a fundamental shift in clinical thinking, moving beyond risk factor control to actively targeting remission. The core perspective is built on the following principles:

Remission as a primary endpoint: This perspective advocates for the recognition of sustained biochemical remission (e.g., normal HbA1c and fasting glucose for at least 3 months without medication) as a valid and primary clinical endpoint in prediabetes care, not merely an incidental outcome.

Diet quality as the central intervention: The authors posit that diet quality, specifically its inflammatory potential, is the most scalable and fundamental therapeutic target for achieving remission.

The DII as a guiding metric: This article proposes the DII as a practical tool to operationalize this concept, helping clinicians and patients assess and modify the inflammatory burden of their dietary choices.

Leveraging public health guidelines: It is suggested that existing evidence-based dietary guidelines, such as the DGA, can be leveraged as a population-level framework for implementing low-inflammatory eating patterns.

A call for causal research: Finally, this article explicitly calls for future randomized controlled trials designed to test DII-guided interventions, moving beyond the current evidence base, which is largely observational or derived from *post-hoc* analyses.

## The case for remission: shifting from delay to reversal

Mounting evidence now challenges the traditional view of prediabetes as an inevitable precursor, firmly establishing remission as an achievable therapeutic goal. Remission, marked by normalization of fasting glucose (<100 mg/dL), 2-hour glucose post-oral glucose tolerance test (<140 mg/dL), and HbA1c (<5.7%), not only halts diabetes onset but also confers lasting benefits, including reduced cardiovascular risk and potential oncologic protection (3, 9, 10). While evidence from type 2 diabetes remission studies (e.g., bariatric surgery and GLP-1 receptor agonists) provides valuable mechanistic analogies, these findings are not directly equivalent to prediabetes remission. Some epidemiological studies suggest broader benefits, such as potential oncologic protection, the evidence for this in the specific context of prediabetes remission remains preliminary and requires further investigation.

It is important to acknowledge that remission definitions vary considerably across trials and publications. Some studies define remission using HbA1c alone (<5.7% or <6.0%), others require normalization of fasting glucose or 2-hour glucose on oral glucose tolerance testing, and few specify a minimum duration of sustained normoglycemia (11). This heterogeneity complicates cross-trial comparisons and the synthesis of evidence. In this article, we adopt the stringent ADA-aligned definition of biochemical normalization (fasting glucose <100 mg/dL, 2-hour glucose <140 mg/dL, and HbA1c <5.7%) without glucose-lowering medications for at least 3 months (12), recognizing that sustained remission is a more robust clinical endpoint than transient biochemical improvement.

Importantly, prediabetes remission offers a promising strategy to curb the escalating global burden of T2D by more effectively preventing progression than standard weight loss goals alone (1). For instance, lifestyle interventions achieving modest weight loss have yielded remission rates of 20-80%, averting T2D by up to 73% over subsequent years (3). Yet, the key to sustained remission lies in addressing inflammation as the unifying pathological link. Chronic inflammation exacerbates insulin resistance, β-cell impairment, and even neuroinflammatory processes, underscoring the need for targeted strategies (6). Anti-inflammatory diets emerge as a primary tool, modulating these pathways to facilitate remission more effectively than generic calorie restriction alone.

## Anti-inflammatory diets as the core mechanism for remission

**Figure 1:**
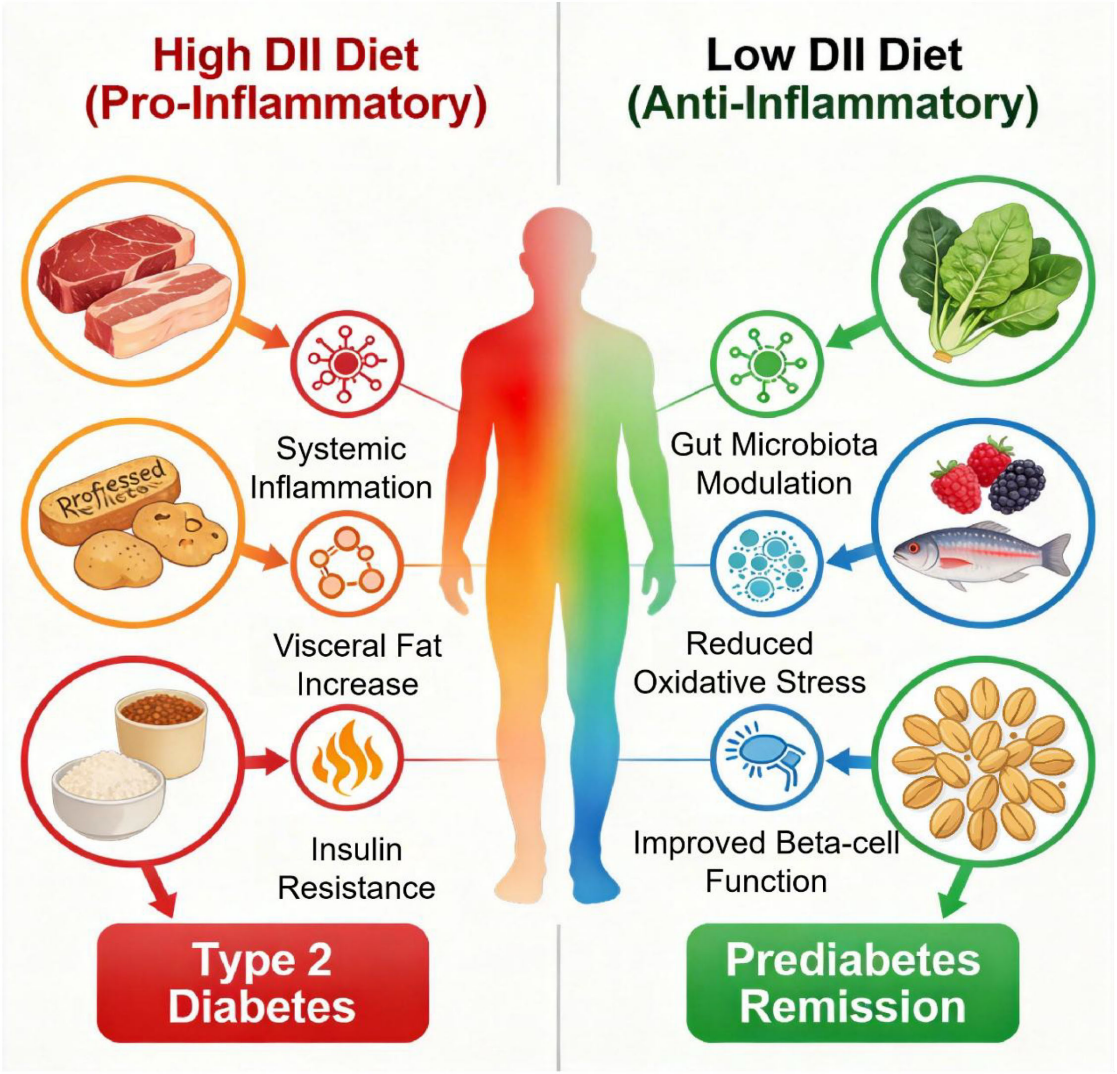
Mechanistic contrast between pro-inflammatory (high-DII) and anti-inflammatory (low-DII) dietary patterns.

Dietary patterns exert profound influence on prediabetes trajectories through their inflammatory potential, as measured by the DII, where positive scores correlate with elevated systemic inflammation markers like C-reactive protein and accelerated inflammaging—age-related inflammation that hastens metabolic decline (6, 13). Epidemiological evidence substantiates this link, demonstrating that individuals with higher (more pro-inflammatory) DII scores have significantly increased odds of prediabetes (14). Importantly, DII should be viewed as a practical integrative metric capturing diet-driven inflammatory potential rather than a direct mechanistic determinant of remission. In prediabetes, high-DII diets rich in red and processed meats, refined carbohydrates, and saturated fats promote hepatic steatosis and visceral adipose tissue expansion, impairing insulin signaling and fostering cytokine-driven resistance (e.g., IL-6, TNF-α). In stark contrast, anti-inflammatory (low-DII) dietary patterns intervene precisely at these pathological junctures: plant-based patterns enrich fiber/polyphenols/omega-3s, modulating gut microbiota toward anti-inflammatory metabolites (e.g., short-chain fatty acids like butyrate) (8). Oxidative stress, amplified in prediabetes, is mitigated by antioxidants in fruits/vegetables, preserving β-cell function. As illustrated in **Figure 1**, the inflammatory potential of dietary patterns creates divergent metabolic trajectories in individuals with prediabetes. A high-DII (pro-inflammatory) diet drives disease progression toward type 2 diabetes by exacerbating systemic inflammation, visceral adiposity, and insulin resistance. Conversely, transitioning to a low-DII (anti-inflammatory) diet facilitates clinical remission through favorable modulation of the gut microbiota, attenuation of oxidative stress, and restoration of β-cell function.

This infographic illustrates the opposing effects of dietary inflammatory potential on prediabetes progression or remission. On the left (pink/red panel), a pro-inflammatory diet rich in refined carbohydrates, processed meats, and saturated fats drives increased cytokine production, chronic low-grade systemic inflammation, visceral adipose tissue expansion, and subsequent insulin resistance. These pathways converge centrally toward metabolic deterioration. On the right (green panel), a low-DII anti-inflammatory diet abundant in vegetables, fruits, whole grains, legumes, nuts, and fatty fish provides fiber, which support favorable gut microbiota modulation, reduction of adipose tissue inflammation and oxidative stress, and preservation or restoration of β-cell function, ultimately promoting insulin sensitivity and normal glucose homeostasis.

Clinical evidence consistently supports this mechanistic framework, though it is important to note that most of this evidence is derived from *post-hoc* analyses of trials not originally designed to test the DII hypothesis. A recent randomized clinical trial (15) demonstrated that an anti-inflammatory diet significantly reduced fasting blood sugar (β: -6.42 mg/dL), HbA1c (β: -0.27%), and body weight (β: -1.02 kg) compared to a control diet in individuals with prediabetes. In the PLIS, a lifestyle intervention inducing ≥5% weight loss achieved 43% remission, driven by improved insulin sensitivity and visceral fat reduction—effects attributable to implicit DII lowering via plant-rich calorie restriction (3). Similarly, the Diabetes Prevention Program (DPP) and its long-term follow-up study demonstrated that participants originally randomized to intensive lifestyle intervention had a 24% lower cumulative incidence of type 2 diabetes over 21 years compared to the placebo group (hazard ratio 0.76 [95% CI 0.68 to 0.85]), with anti-inflammatory shifts in adipose tissue playing a central role in the early intervention effects (16). Even without net weight loss, remission occurs through enhanced β-cell responsiveness to GLP-1 and improved sensitivity, as demonstrated in recent trials where plant-based elements likely contributed to gut hormone modulation and microbiota-mediated inflammation resolution (9). Bariatric surgery, yielding 58-86% remission over 4 years, exemplifies this by altering gut hormones (e.g., GLP-1/GIP surges) and microbiota composition, effectively mimicking low-DII dietary impacts (10). Pharmacologic agents like semaglutide parallel these mechanisms, but dietary approaches offer broader accessibility and sustainability (10).

Further trials reinforce the anti-inflammatory pivot. Although a small pilot trial suggested remarkably high remission rates with a specific high-protein diet, underscoring the potential role of incretin modulation, the broader and more consistent evidence points to the superiority of plant-predominant, low-DII patterns for sustainable remission (17). Community-based cohorts have tied reductions in body mass index and body fat percentage to remission, with underlying gut-mediated anti-inflammatory effects evident (18). In an Indian trial focused on women with isolated impaired fasting glucose, lifestyle modifications achieved 45.9% remission, highlighting how fiber-rich interventions enhance insulin sensitivity via microbiota shifts (19). Multinational data from the PREVIEW trial echoed this, with moderate-protein, moderate-glycaemic index diets yielding 20.6-26.3% remission rates over 3 years, independent of weight changes and associated with reduced inflammaging markers (20). Supplementation studies, such as those with insoluble oat fiber, have shown glycemic improvements in prediabetes, underscoring fiber’s role in anti-inflammatory metabolite production, albeit with limited cognitive gains (21). Collectively, from lifestyle interventions to bariatric surgery, the common thread underpinning successful remission is the attenuation of diet-driven chronic inflammation and its downstream metabolic consequences. **Table 1** summarizes key trials reporting prediabetes remission and their dietary features.

**Table 1:** Key trials reporting prediabetes remission and dietary features.

Trial name	Population	Intervention	Remission definition	Remission rate	Key mechanistic insight
Prediabetes Lifestyle Intervention Study (PLIS) (3)	Adults with prediabetes (impaired fasting glucose, impaired glucose tolerance, or both; multicenter in Germany; n=1,105 originally, *post-hoc* analysis on those achieving ≥5% weight loss)	Intensive lifestyle intervention including dietary counseling (reduce total fat <30% energy, saturated fat <10%, increase fiber >15g/1,000 kcal) and physical activity to achieve ≥5% weight loss	Return to normal glucose regulation: fasting glucose <100 mg/dL (<5.6 mmol/L), 2-h glucose <140 mg/dL (<7.8 mmol/L), HbA1c <5.7% (<39 mmol/mol) without glucose-lowering medication	43% at 12 months	Improved whole-body insulin sensitivity and reduced visceral adipose tissue (VAT) mass; no significant changes in insulin secretion, liver fat, or pancreas fat
High-Protein Diet Trial (17)	Adults with prediabetes (small pilot study; n=24 randomized to HP vs. HC diets)	High-protein diet (30% energy from protein, 30% fat, 40% carbohydrate) for 6 months, with calorie restriction for weight loss	Normalization of glucose levels (fasting glucose <100 mg/dL, 2-h glucose <140 mg/dL, HbA1c <5.7%) without medication	100% at 6 months (vs. 33% in high-carbohydrate control group)	Increased GLP-1 and GIP levels leading to improved insulin sensitivity and β-cell function; reduced ghrelin (hunger suppression); improvements in cardiovascular risk factors, metabolic parameters, and oxidative stress
Community-Based LSM Trial in India (19)	Women with isolated impaired fasting glucose (i-IFG) in Kerala, India (community-based; n=1,092 randomized, 1,080 completed)	Structured lifestyle modification including dietary advice (increase fiber-rich foods, reduce refined carbs and fats) and physical activity promotion for 12 months	Return to normoglycemia: fasting plasma glucose <100 mg/dL without medication (HbA1c and OGTT not required in all cases)	45.9% at 12 months (vs. 6.3% in control)	Enhanced insulin sensitivity via microbiota shifts from fiber-rich diet; reductions in HOMA-IR, weight, waist circumference, and fat mass; improvements in cardiometabolic health
PREVIEW Trial (20)	Adults with overweight/obesity (BMI ≥25 kg/m²) and prediabetes (fasting glucose 5.6–6.9 mmol/L and/or 2-h glucose 7.8–11.0 mmol/L; multinational; n=1,856 in modified ITT)	Initial 8-week low-energy diet (3,400 kJ/day) for weight loss, followed by 3-year ad libitum maintenance with either moderate-protein moderate-GI (15% protein, GI >56, 55% carbs emphasizing whole grains, vegetables, fruits) or high-protein low-GI (25% protein, GI <50, 45% carbs emphasizing low-GI cereals, legumes, low-fat dairy, fish) diets plus physical activity	Return to normal fasting glucose <5.6 mmol/L and normal glucose tolerance (2-h glucose <7.8 mmol/L)	20.6% (moderate-protein moderate-GI) to 26.3% (at 1 year) overall; at 3 years: 20.6% (moderate-protein moderate-GI) vs. 15.5% (high-protein low-GI)	Remission independent of weight changes; associated with greater reductions in BMI and fat mass; reduced inflammaging markers; moderate-protein moderate-GI diet superior for sustained remission
Pakistan Diabetes Prevention Trial (22)	Adults with prediabetes in Pakistan (community-based; specific n not detailed, but culturally diverse urban/rural settings)	Culturally tailored lifestyle changes including dietary modifications (increase plant-based foods, fiber, reduce processed foods and sugars) and physical activity for diabetes prevention	Normalization of glucose levels: fasting glucose <100 mg/dL, 2-h glucose <140 mg/dL, HbA1c <5.7% without medication	62% (time point not specified, likely at study end ~12–24 months)	Gut-mediated anti-inflammatory effects from localized, fiber-rich interventions; improvements in body mass index and body fat percentage; scalable for low-resource settings
Anti-Inflammatory Diet RCT (15)	Adults with prediabetes (open-label RCT, Iran; n=98 randomized)	Anti-inflammatory diet (75% calorie intake with emphasis on fruits, vegetables, whole grains, polyphenols, omega-3; low pro-inflammatory foods) vs control healthy diet for 12 weeks	Not primary endpoint (focus on cardio-metabolic improvement toward normoglycemia)	Anti-inflammatory arm showed superior effects: fasting blood glucose ↓6.42 mg/dL, HbA1c ↓0.27%, body weight ↓1.02 kg (vs control)	Direct attenuation of diet-driven inflammation leading to best glycemic control and cardiometabolic improvements

Remission can be achieved through multiple independent or overlapping pathways, including weight loss, energy restriction, reduction in hepatic and visceral fat, favorable incretin changes, and improvements in insulin sensitivity. Most of the trials cited here (e.g., PLIS, PREVIEW, DPP) were not originally designed to test the DII hypothesis; rather, DII associations are often derived from *post-hoc* or observational analyses. Therefore, while low-DII patterns show mechanistic promise, they represent one important dimension among several contributors to glycemic restoration.

## Practical implementation: leveraging the 2025–2030 dietary guidelines

Translating these mechanisms into practice requires accessible frameworks, and the 2025–2030 Dietary Guidelines for Americans (released in 2026) provide precisely that—an evidence-based blueprint for anti-inflammatory eating. The 2025–2030 Dietary Guidelines for Americans (DGA) provide an influential and evidence-based blueprint for healthy eating that aligns strongly with a low-inflammatory dietary pattern, although it is not specifically designed for prediabetes remission. Notably, the guidelines operationalize a low-DII eating pattern through specific, actionable recommendations: prioritizing whole, minimally processed plant foods like vegetables, fruits, whole grains, and legumes (e.g., at least 5–9 servings of fruits/vegetables daily and 2–4 servings of whole grains, adjusted by calorie needs), while limiting ultra-processed foods to curb added sugars (recommended <10% of calories, with stronger per-meal guidance in some interpretations), sodium, and saturated fats—directly aligning with DII reduction^1^. The guidelines emphasize nutrient-dense proteins from plant sources, nuts, and fatty fish to boost omega-3 intake, alongside fiber goals (38 g/day for men, 25 g/day for women) that support gut health and inflammation control.

This framework seamlessly integrates with remission strategies. Reducing ultra-processed foods lowers DII, mirroring successes in PLIS and DPP where plant-centric restrictions enhanced insulin sensitivity. The plant-predominant focus fosters microbiota modulation, as seen in PREVIEW’s moderate-protein, moderate-GI arms achieving superior remission (20.6%) versus high-protein, low-GI (15.5%) (20). Cultural tailoring in the guidelines addresses equity, enabling adaptations in low- and middle-income settings, such as Pakistan’s diabetes prevention trial where localized interventions yielded 62% remission (22). Beyond individual dietary advice, the guidelines’ push for screening and counseling institutionalizes the goal of remission within primary care, marking a critical step from theory to population health impact (23). Implementation tools like glucometers for self-monitoring can overcome barriers in underserved areas, ensuring broad applicability (24). For clinicians seeking to implement this approach, we propose the following clinic-ready checklist (**Table 2**). These recommendations are derived from the DGA and key trials, but should be adapted to local food availability and cultural preferences.

**Table 2:** Clinic-ready checklist for implementing a low-inflammatory diet.

Action item	Specific recommendation	Rationale and expected impact on inflammation and remission
Replace sugar-sweetened beverages	With water or unsweetened tea/coffee	Eliminates major sources of added sugars and ultra-processed components, directly lowering DII scores and reducing postprandial glucose spikes and systemic inflammation (e.g., CRP)
Increase legume consumption	At least 4 times per week (e.g., beans, lentils, chickpeas)	Provides soluble fiber and polyphenols that promote short-chain fatty acid production, improve gut microbiota composition, and enhance insulin sensitivity
Include fatty fish	Twice weekly (e.g., salmon, mackerel, sardines)	Supplies EPA/DHA omega-3 PUFAs that suppress pro-inflammatory cytokines (IL-6, TNF-α) and support resolution of chronic low-grade inflammation
Consume unsalted nuts	A small handful (approx. 30 g) daily	Delivers monounsaturated fats, fiber, and antioxidants that reduce oxidative stress and visceral adipose tissue inflammation
Increase total dietary fiber	Meet DGA targets (22–28 g/day for women, 28–34 g/day for men, depending on age)	Strengthens intestinal barrier function, generates anti-inflammatory metabolites (e.g., butyrate), and facilitates sustained glycemic improvement toward remission
Limit ultra-processed foods	Minimize as primary contributors to added sugars, sodium, and unhealthy fats	Core strategy to achieve and maintain a low-DII dietary pattern, mirroring the plant-rich interventions associated with higher remission rates in PLIS, PREVIEW, and community trials

Adapted from the 2025–2030 dietary guidelines for Americans and evidence from key prediabetes remission trials (PLIS, PREVIEW, Pakistan Diabetes Prevention Trial). Targets should be individualized based on total caloric needs, cultural preferences, and comorbidities.

## Conclusion and outlook

Anti-inflammatory dietary patterns stand as the pivotal mechanism and pathway for prediabetes remission, disrupting inflammatory cascades from gut to β-cells and offering a scalable route to metabolic restoration. By harnessing DII-guided strategies—bolstered by the 2025–2030 Dietary Guidelines’ emphasis on whole plant foods and ultra-processed food limits—this approach redefines prediabetes management. While challenges like long-term adherence persist, with relapse rates up to 58% by year 4 tied to weight regain and inflammaging, future directions lie in precision nutrition: stratifying by microbiota or inflammaging markers (e.g., sCD14) and integrating omics data to personalize interventions (25–27).

However, it is critical to acknowledge the limitations inherent in the current evidence base. This article does not present a systematic review of the literature; such a formal review would be required to comprehensively assess the heterogeneity of inflammatory markers used across studies (e.g., hs-CRP, IL-6, TNF-α). DII serves as a validated composite dietary exposure score rather than a direct biological endpoint. Future research should prioritize pairing DII with a panel of multiple inflammatory markers—including CRP, IL-6, and gut-derived metabolites—to better elucidate the mechanistic pathways linking diet to remission. Furthermore, the link between DII and prediabetes remission, while strongly suggestive, is largely based on *post-hoc* analyses and observational inferences from trials not originally designed to test DII-guided interventions—a causal relationship has not been definitively proven. The heterogeneity of remission definitions across trials, with some using HbA1c alone, others requiring OGTT, and few specifying a minimum duration of sustained normoglycemia, makes cross-trial comparisons challenging, and it must be acknowledged that remission is not always permanent, with relapse often linked to weight regain. Additionally, the DII itself, while validated, does not capture all aspects of diet quality, such as food processing (NOVA classification), glycemic load, or meal timing, and its calculation may not be easily transferable to all cultural contexts without adaptation of food composition databases. Finally, the mechanistic pathways discussed, while plausible, are often broad, and the specific contribution of each pathway to remission in an individual patient remains difficult to quantify.

Consequently, future research must prioritize randomized controlled trials that directly test the efficacy of DII-based dietary prescriptions for remission. Precision nutrition approaches, stratifying individuals by baseline inflammatory status, microbiota composition, or genetic risk, hold promise for personalizing interventions and improving long-term adherence (28). While challenges like long-term adherence and the need for more direct evidence remain, the current scientific foundation is robust enough to begin integrating this approach into clinical practice and public health policy today. We therefore reiterate our call for a definitive paradigm shift, where inducing and maintaining remission through high-quality, anti-inflammatory nutrition becomes a primary objective in prediabetes care—not merely as a metabolic correction, but as a foundation for healthier aging.

## Footnotes

1 U.S. Department of Agriculture and U.S. Department of Health and Human Services. Dietary Guidelines for Americans, 2025-2030. Washington, DC: U.S. Government Printing Office; 2026. Available from: https://www.dietaryguidelines.gov/
